# Molecular identification of head lice collected in Franceville (Gabon) and their associated bacteria

**DOI:** 10.1186/s13071-020-04293-x

**Published:** 2020-08-11

**Authors:** Celia Scherelle Boumbanda-Koyo, Oleg Mediannikov, Nadia Amanzougaghene, Sandrine Lydie Oyegue-Liabagui, Roméo Karl Imboumi-Limoukou, Didier Raoult, Jean Bernard Lekana-Douki, Florence Fenollar

**Affiliations:** 1 Aix Marseille Univ, IRD, AP-HM, SSA, VITROME, Marseille, France; 2 IHU-Méditerranée Infection, Marseille, France; 3 Unité d’Evolution, Epidémiologie et Résistances Parasitaires (UNEEREP), Centre International de Recherches Médicales de Franceville (CIRMF), B.P. 769 Franceville, Gabon; 4 Ecole Doctorale Régionale en Infectiologie Tropicale d’Afrique Centrale, B.P. 876 Franceville, Gabon; 5 Aix Marseille Univ, IRD, AP-HM, MEPHI, Marseille, France; 6 Département de Parasitologie-Mycologie et Médecine Tropicale, Faculté de Médecine, Université des Sciences de la Santé (USS), B.P. 4009 Libreville, Gabon

**Keywords:** Head lice, *Pediculus humanus*, *Acinetobacter* spp., *Borrelia* spp., Gabon

## Abstract

**Background:**

*Pediculus humanus*, which includes two ecotypes (body and head lice), is an obligate bloodsucking parasite that co-evolved with their human hosts over thousands of years, thus providing a valuable source of information to reconstruct the human migration. Pediculosis due to head lice occurred each year throughout the world and several pathogenic bacteria, which are usually associated with body lice, are increasingly detected in them. In Gabon, where this pediculosis is still widespread, there is a lack of data on genetic diversity of head lice and their associated bacteria.

**Methods:**

This study aimed to investigate the phylogeny of head lice collected in Gabon and their associated bacteria, using molecular tools. Between 26 March and 11 April 2018, 691 head lice were collected from 86 women in Franceville. We studied the genetic diversity of these lice based on the cytochrome *b* gene, then we screened them for DNA of *Bartonella quintana*, *Borrelia* spp., *Acinetobacter* spp., *Yersinia pestis*, *Rickettsia* spp., *R. prowazekii*, *Anaplasma* spp. and *C. burnetii*, using real time or standard PCR and sequencing.

**Results:**

Overall 74.6% of studied lice belonged to Clade A, 25.3% to Clade C and 0.1% to Clade E. The phylogenetic analysis of 344 head lice yielded 45 variable positions defining 13 different haplotypes from which 8 were novel. Bacterial screening revealed the presence of *Borrelia* spp. DNA in 3 (0.4%) of 691 head lice belonging to Clade A and infesting one individual. This *Borrelia* is close to *B. theileri* (GenBank: MN621894). *Acinetobacter* spp. DNA has been detected in 39 (25%) of the 156 screened lice; of these 13 (8.3%) corresponded to *A. baumannii. Acinetobacter nosocomialis* (*n* = 2) and *A. pittii* (*n* = 1) were also recorded.

**Conclusions:**

To of our knowledge, this study is the first to investigate the genetic diversity of head lice from Gabon. It appears that Clade C is the second most important clade in Gabon, after Clade A which is known to have a global distribution. The detection of *Borrelia* spp. DNA in these lice highlight the potential circulation of these bacteria in Gabon.
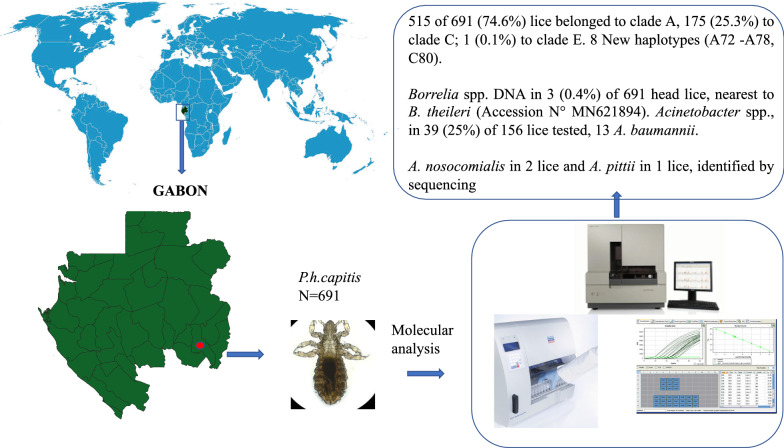

## Background

Archaeological studies have shown that lice are very old human parasites [1]. Indeed, lice and their nits had been found on hair remains from an archaeological site in Brazil dating back to 8000 years BC, on the hair of an individual living in the cave of Nahal Hemar in Israel, dating back to 9000 years BC [2]. More recently, lice and louse nits had been found in Hatzeva in the Judean desert, in Moa, and around the Dead Sea (Arava), on combs dating back 2000 years [3]. These findings provide ample evidence that lice have parasitized humans for a very long time and have probably completed their migration “Out of Africa” through the migration of their human host [1]. Therefore, lice could be a good biological marker to understand human migration and evolution [4]. Lice are host specific; only two genera of lice infest humans: the genus *Pthirus* with a unique representative *Pthirus pubis* (crab lice) and the genus *Pediculus* [5]. The latter is of concern and contains two ecotypes: *Pediculus humanus capitis* (head lice) which lives and thrive on scalp and hair and *P. h. humanus* (body lice) which lives and thrive on skin and clothes [6].

Phylogenetic studies based on mitochondrial genes, mainly cytochrome *b* (*cytb*) and cytochrome *c* oxidase subunit 1 (*cox*1), have permitted to split lice into six divergent clades, five of which (A to E) are well known and one named clade F, which has recently been discovered [7]. Head lice encompass the full genetic diversity of clades, while body lice belong only to clades A and D [8]. Clade A has a global distribution and is the most prevalent [9, 10]. Clade B is frequently found in Americas, Europe and Australia, but has recently been detected in South Africa, Saudi Arabia and Algeria [8, 11]. Clade C was found in Ethiopia, Congo-Brazzaville, Nepal, Pakistan and Thailand [7, 12]. Clade D was found in the Democratic Republic of the Congo (DR Congo), the Republic of the Congo (Congo-Brazzaville), Ethiopia and Zimbabwe [6, 7, 12, 13]. Clade E has been described in Senegal, Mali, DR Congo and found among Nigerian refugees in Algeria and migrant communities living in Bobigny, France [14–17].

In addition to their role as markers of human migration, lice can be a significant health hazard. Body lice are able to transmit pathogenic bacteria such as *Bartonella quintana* (the agent of trench fever, bacillary angiomatosis, chronic lymphadenopathies and endocarditis) [18], *Borrelia recurrentis* (the causative agent of louse-borne relapsing fever) [19] and *Rickettsia prowazekii* (responsible for epidemic typhus) [20]. The role of head lice as vector of these bacteria is still suggestive. Head lice have been shown to have a stronger immune response than body lice, resulting in the rapid elimination of ingested bacteria [21]. However, studies on lice collected worldwide indicate the presence of a large number of pathogenic bacteria, including, *Acinetobacter* spp. in head lice collected in France, Algeria [22], Republic of the Congo and DR Congo [15, 16, 22, 23]. *Bartonella quintana* had been found in head lice collected in Madagascar, Senegal [24], in Nepal [25], the USA [26], and on head lice nits of a homeless person in Marseille [27]. *Coxiella burnetii* had been detected in head lice collected in Mali, Algeria and France [17, 28, 29]. *Borrelia recurrentis* and *B. theileri* had been found on head lice of pygmies from the Republic of the Congo [12]. *Yersinia pestis* was detected in head lice collected from persons living in a highly plague-endemic area in DR Congo [13]. *Rickettsia aeschlimannii*, as well as the DNA of potential new species from the genera *Anaplasma* and *Ehrlichia* were also detected in head lice [17]. Moreover, under experimental conditions head lice are able to acquire, maintain and transmit *R. prowazekii* and *B. quintana* [21]. Since head lice are able to carry bacteria, they could thus passively transmit them to their host, by excreting them on the injured skin of the host. Since pediculosis due to head lice is widespread throughout the world [30], controlling their associated pathogens is important to prevent the potential outbreaks related to these pathogens.

In Gabon, pediculosis caused by head lice is very prominent but poorly reported, because its leads to stigmatization of the carriers. To the best of our knowledge, this is the first study investigating the genetic diversity of head lice from Gabon and their associated bacteria.

## Methods

### Lice collection and DNA extraction

**Fig. 1:**
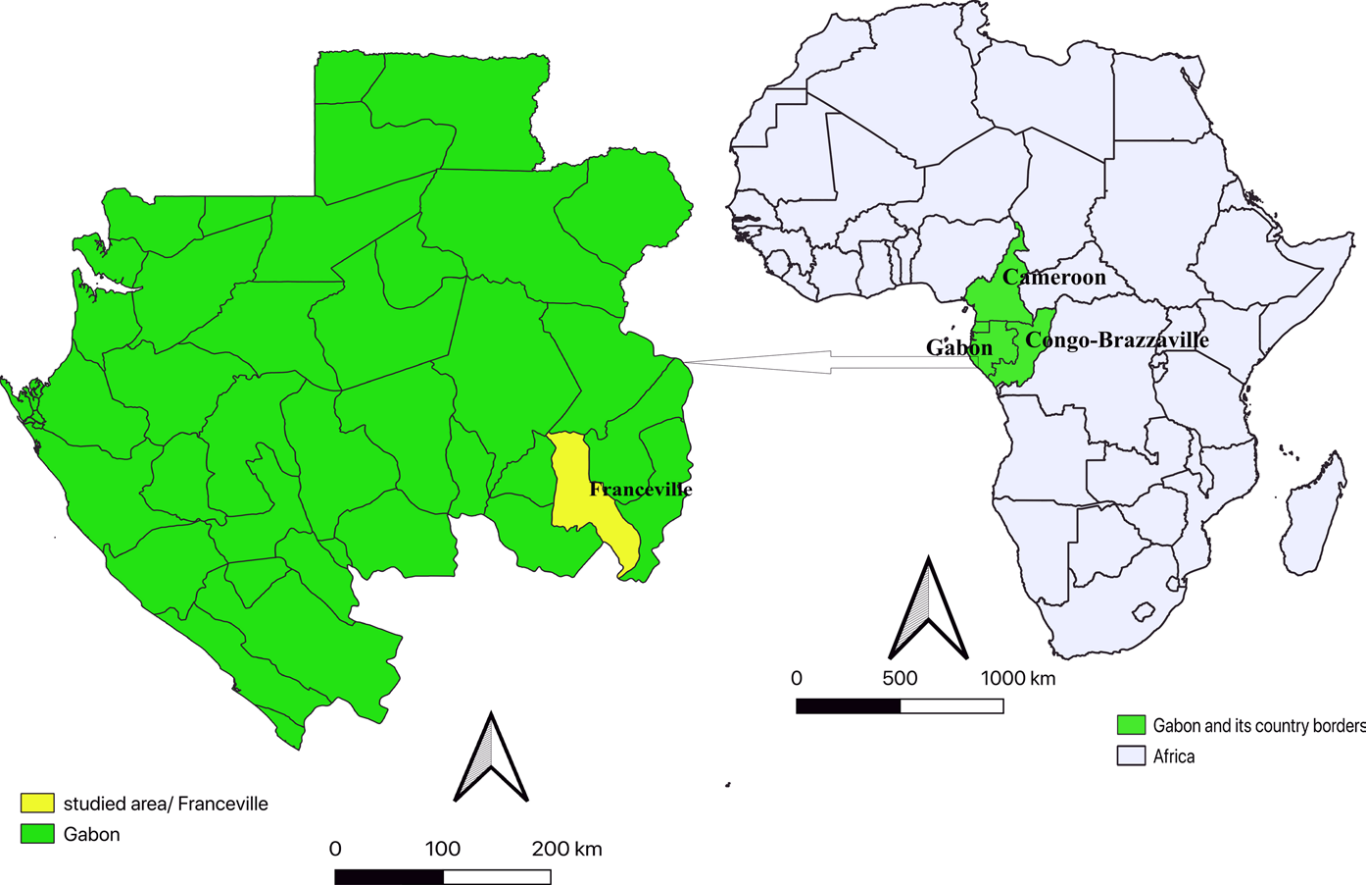
Map of Africa with location of Gabon and study area in Gabon

Between March 26th and April 11th, 2018, the staff of the Centre International de Recherche Médicales de Franceville (CIRMF) harvested lice from inhabitants of Franceville town (Fig. 1), in the department of M’Passa. Eighty-eight apparently healthy women were recruited in 11 very close neighborhoods of Franceville city. These women were thoroughly examined for head lice and a total of 691 lice were collected. For each individual, lice were collected in a Falcon tube containing 70% ethanol which was then stored at -20 °C at CIRMF, before being sent to IHU Mediterranée-Infection (Marseille, France) for further analysis.

Once in Marseille, each louse was analyzed individually. To overcome any false positives due to environmental contamination, the lice were disinfected by immersion in ethanol for 5 min and rinsed twice by immersion in sterile distilled water. Then, the lice were dried and cut in half, if possible; small individuals were treated whole. A prelysis of lice was performed in 200 μl of buffer G2 and 10 μl Proteinase K supplied in the Qiagen DNA Tissue kit (Qiagen, Courtaboeuf, France), after being crushed with a scalpel blade. DNA extraction was automatically performed in the BioRobot EZ1 Advanced XL instrument (Qiagen); the elution volume was 100 μl. DNA quantification was carried out using a NanoDrop ND-1000 (Thermo Fisher Scientific, Waltham, MA, USA). DNA was stored at − 20 °C until the start of analysis.

### Genotyping lice by real-time quantitative PCR (qPCR)

Lice clades were initially determined by qPCR using the A-D and B-C/E duplex primers (Additional file 1: Table S1) [15, 17]. For each qPCR run, the final volume of 20 μl was composed of 10 μl of the Roche master mix (Roche Applied Science, Mannheim, Germany), 3 μl of water, 0.5 μl of each primer and probe, 0.5 μl of UDG (uracil DNA glycosylase) and 5 μl lice genomic DNA. Amplification was performed in a CFX96 Real-Time PCR detection system (Bio-Rad Laboratories, Foster City, CA, USA) according to the following amplification parameters: one step at 50 °C for 2 min, an initial denaturation at 95 °C for 5 min, followed by 40 cycles of 95 °C for 15 s and 60 °C for 30 s for annealing extension.

### Determination of clades by standard PCR coupled with sequencing

For better characterization and identification of possible new haplotypes, we amplified and sequenced a 347-bp fragment of the cytochrome *b* gene for 344 randomly selected samples and for lice where clade determination by qPCR was not successful. The final reaction volume of 25 µl consisted of 12.5 µl of Amplitaq gold master mix, 0.75 µl of each primer (20 µM), 6 µl of water and 5 µl of genomic DNA. Amplification was performed in a Peltier PTC-200 model thermal cycler (MJ Research Inc, Watertown, MA, USA) according to the following parameters: one step of incubation at 95 °C for 15 min, 40 cycles of 1 min at 95 °C, 30 s at 56 °C and 1 min at 72 °C, followed by a final extension step at 72 °C for 5 min. PCR results were visualized on a 1.5% agarose gel and stained with SYBR Safe (Invitrogen, San Diego, CA, USA) under transilluminator UV light. The PCR amplicons were then purified using the Macherey Nagel NucleoFast 96 PCR plate (Macherey-Nagel, Hoerdt, France), following the manufacturer’s instructions. Once purified, PCR products were sequenced using the big Dye Terminator Sequencing kit (Perkin Elmer Applied Biosystems, Foster City, CA, USA) using a 3500XL Genetic Analyzer (Thermo Fisher Scientific). The sequences obtained were analyzed with ChromasPro software (ChromasPro 1.7, Technelysium Pty Ltd., Tewantin, Australia).

### Screening of bacterial DNA in head lice

In order to screen louse-borne pathogens, lice DNA was pooled in 68 pools of 10 lice (10 µl of each louse) and 1 pool of 11 head lice. A total of 69 DNA pools were screened for the presence of DNA from *B. quintana*, *Borrelia* spp., *Acinetobacter* spp., *Y. pestis*, *Rickettsia* spp., *R. prowazekii*, *Anaplasma* spp. and *C. burnetii.* The DNA was screened using qPCRs as previously described [15]. We created a DNA pool with 10 samples of 10 µl each. In the pools, each sample was therefore in a proportion of 1 Log of its initial concentration. We therefore raised the number of cycles from 40 Cq to 45 Cq to ensure we did not miss any potentially positive samples in the pools. A pool consisted of 10 consecutive samples in our database, and the 11 remaining samples were pooled together. The ready to use Master mix Light Cycler 480 Probes (Roche Applied Science) was used in the PCR reactions carried out in a CFX96 Real-Time PCR detection system. Genomic DNA of targeted bacteria was used as positive controls and the master mix was used as a negative control in each PCR run. The amplification of DNA was performed according to the following parameters: one step of incubation at 50 °C for 2 min (for UDG activation), a step of 95 °C for 5 min for initial denaturation and 45 cycles of 5 s at 95 °C and 30 s at 60 °C. When a pool was found positive in a qPCR with a quantification cycle (Cq) ≤ 38, lice of this pool were tested individually. A cycle threshold for an individual louse was considered at Cq ≤ 35. Samples positive by qPCR for *Acinetobacter* spp. were subjected to qPCR specific for *A. baumannii*, as previously reported [31]. For those negative for *A. baumannii* but positive for *Acinetobacter* spp., we sequenced the *rpoB* gene, as previously described [32]. Samples positive by *Borrelia* spp. qPCR were studied in a standard PCR targeting the *flaB* gene using primers designed for nested PCR [33].

### Data analyses

The electropherograms obtained were assembled and edited using ChromasPro 1.7 software (Technelysium Pty Ltd, City, Country). The corrected sequences were then analyzed using BLAST (www.ncbi.nlm.nih.gov/blast/Blast.cgi) and compared with sequences in the GenBank database. ClustalW alignments were performed in MEGA6 [34]. Haplotypes were identified using DnaSP v5.10 software [35]. The phylogenetic tree was inferred using the Maximum Likelihood method based on the Tamura 3-parameter model for nucleotide sequences under 500 bootstrap replicates.

## Results

### Genotyping of lice clades

A total of 86 women reported to have head lice were recruited in Franceville from which 691 head lice were collected. Phylogenetic molecular clade identification (qPCR and *cytb* amplification/sequencing) analysis showed that 515 of 691 (74.6%) analyzed lice belonged to clade A, 175 (25.3%) belonged to clade C and 1 (0.1%) belonged to clade E (Table 1).

**Table 1:** Single and co-infestations of people with lice of different lice clades

Clade	No. of people infested (%) (*N* = 86)	No. of lice (%) (*N* = 691)	Bacteria detected by clade (frequency)
Single infestation	81 (94.2)	–	
Clade A	73 (84.9)	515 (74.6)	*A. baumannii* (*n* = 9), *A. nosocomialis* (*n* = 2), *A. pittii*, *Borrelia* spp. (*n* = 3)
Clade C	8 (9.3)	175 (25.3)	*A. baumannii* (*n* = 4)
Clade E	0	1 (0.1	
Co-infestation	5 (5.8)	–	
Clade A + C	4 (4.6)	–	
Clade A + E	0	–	
Clade C + E	0	–	
Clade A + C + E	1 (1.2)	–	

**Fig. 2:**
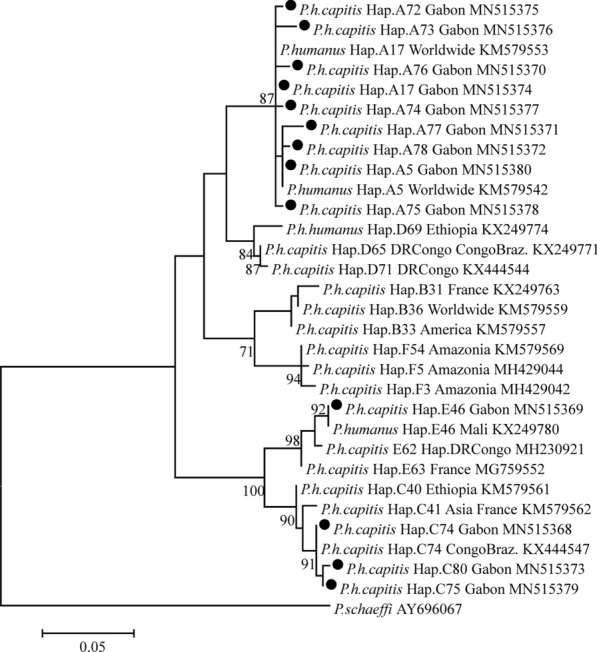
Molecular phylogenetic analysis of *cytb* sequences of lice from Gabon. The evolutionary history was inferred using the Maximum Likelihood method based on the Tamura 3-parameter model [34]. The tree is drawn to scale, with branch lengths measured in the number of substitutions per site. The analysis involved 27 nucleotide sequences. Codon positions included were 1st + 2nd + 3rd + noncoding. There was a total of 272 positions in the final dataset. Evolutionary analyses were conducted in MEGA7. Haplotypes marked by a black circle are those identified in this study

For the phylogenetic study, a partial *cytb* gene (347 bp) of 344 head lice was sequenced. *cytb* sequence analysis yielded 45 variable positions defining 13 different haplotypes (Fig. 2) from which 8 were novel (Table 2). Of these, 325 (94.5% of 344) belonged to clade A, from which 306 sequences corresponded to haplotype A17 and 11 corresponded to haplotype A5. The 8 remaining clade A sequences corresponded to new haplotypes which are named here A72 (1 sequence), A73 (1 sequence), A74 (1 sequence), A75 (1 sequence), A76 (1 sequence), A77 (1 sequence) and A78 (2 sequences). Eighteen sequences (20.9% of 344) were clade C, from which 13 sequences corresponded to haplotype C74, 4 to haplotype C75 and one was a novel haplotype named here C80. One louse showing a very low amplitude melting curve with a clade C probe was identified as a member of clade E by sequencing and corresponded to haplotype E46. All the new haplotypes found in this study were deposited in the GenBank database under the accession numbers MN515370-MN515373 and MN515375-MN515380.

**Table 2:** Identified lice clades and corresponding haplotypes

Clade of lice	Haplotype	*n*	GenBank ID
Clade A (*n* = 325)	A17	306	MN515374
	A5	11	MN515380
	**A72**	1	**MN515375**
	**A73**	1	**MN515376**
	**A74**	1	**MN515377**
	**A75**	1	**MN515378**
	**A76**	1	**MN515370**
	**A77**	1	**MN515371**
	**A78**	2	**MN515372**
Clade C (*n* = 18)	C75	4	MN515379
	C74	13	MN515368
	**C80**	1	**MN515373**
Clade E (*n* = 1)	E46	1	MN515369

*Note*: The haplotypes in bold are the new haplotypes identified in this study

### Single and multiple infections with lice of different clades

Among the 86 women sampled, 73 (84.9%) were infested with lice belonging to clade A only, and 8 (9.3%) were infested with lice of clade C only. Four (4.6%) of the 86 individuals were concomitantly infested with lice belonging to clade A + C, and one (1.2%) was infested with lice belonging to three clades (A + C + E). Only one clade E louse was identified in our study. Thus, no co-infection with clades A + E or C + E was observed.

### Identification of head lice pathogens

**Fig. 3:**
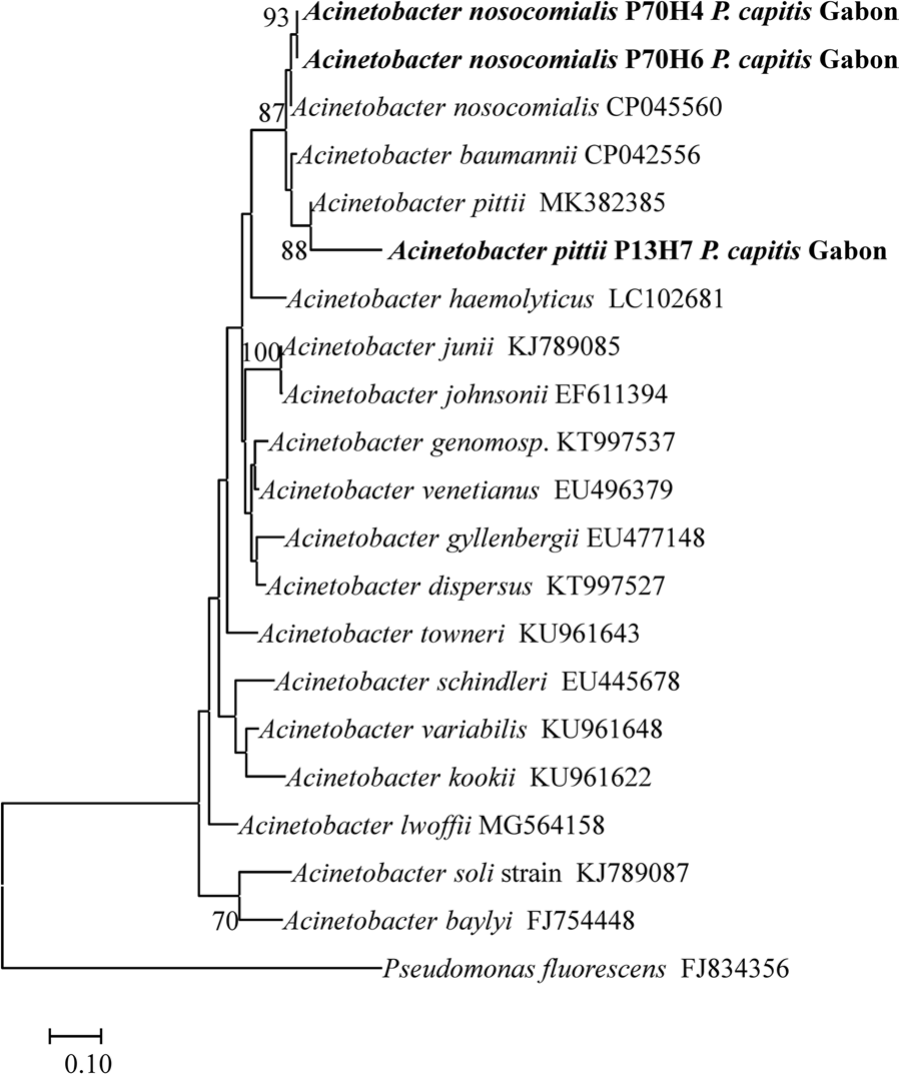
Molecular phylogenetic analysis of *rpoB* sequences of *Acinetobacter* spp. The *rpoB* sequences of *Acinetobacter* spp. were aligned using the CLUSTALW method and evolutionary analyses were conducted in MEGA7 software using the Maximum Likelihood method based on the Tamura-Nei model. There was a total of 262 positions in the final dataset. Species in bold are those identified in this study

We searched the DNA of 7 pathogenic bacteria out of a total of 691 head lice collected. Among all screened bacterial genera, *Acinetobacter* spp. were found in 29 of the 69 DNA pools. Of the 290 lice belonging to these 29 pools, we screened 156 head lice for which DNA was available. Thirty-nine of the 156 (25%) screened lice were positive for *Acinetobacter* spp. Among them, 13 out of 39 (33.3%) lice infesting 8 patients were positive for *A. baumannii*. The number of lice infesting the 8 individuals, and the number of lice infested with *A. baumannii* in these individuals, are recorded in Additional file 2: Table S2. Nine lice belonged to clade A and the 4 belonged to clade C. Of the remaining 26 head lice that were positive for the *Acinetobacter* spp. qPCR and negative for *A. baumannii*, only 3 were successfully sequenced. Two of the 3 sequences shared 99.7% and 100% of similarity, respectively, with *A. nosocomialis* (GenBank: CP020588) and the last sequence shared 99% similarity with *A. pittii* (GenBank: CP040911) (Fig. 3). The two lice positive for *A. nosocomialis* infested the same patient and belonged to clade A. The louse harboring *A*. *pittii* also belonged to clade A.

**Fig. 4:**
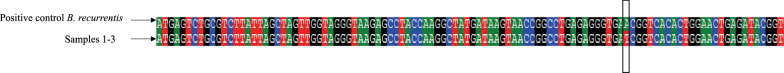
Alignment of the 148-bp fragment of *Borrelia recurrentis 16S* rRNA with those of the two lice positive for this gene using BioEdit software (version 7.2)

We also detected DNA of *Borrelia* spp. in one of the 69 DNA pools. After testing the 10 samples of this pool, 3 samples were positive using qPCR. Sequencing of a 148-bp fragment of the qPCR product targeting *Borrelia 16S* rRNA sequence, allowed us to confirm that these lice were undoubtedly infected with *Borrelia* spp. (Fig. 4). The 3 sequences obtained were 100% identical to each other and the BLAST search of these sequences in GenBank showed that the amplified sequence shared 100% identity with *B. theileri* (GenBank: MN621894). All these 3 positive head lice infested the same individual and belonged to clade A.

No samples were positive for *B. quintana*, *Y. pestis*, *Rickettsia* spp., *R. prowazekii*, *Anaplasma* spp. or *C. burnetii*.

## Discussion

We studied the genetic diversity and associated pathogens of head lice from Gabon. To the best of our knowledge, this is the first study of this kind in Gabon, which investigates both phylogeny of head lice and their associated pathogens. Overall, 691 head lice were collected from 86 female individuals. We found that the studied lice belonged to the three mitochondrial clades, A (74.6%), C (25.3%) and E (0.1%). The presence of clade A in Gabon is not surprising, because this clade is found in all studies investigating the genetic diversity of human lice worldwide, thus proving its worldwide distribution [36]. However, we observed a very low interhaplogroup genetic diversity in the lice studied. Indeed, 84.9% of individuals were infested with head lice belonging to clade A only, while 9.3% of individuals were infested with lice belonging to clade C only. Clade C has already been described in head lice of pygmies (37.3% of 86 screened individuals) from the Republic of the Congo [12], which borders Gabon.

There is a significant migratory flow between Gabon and the Republic of the Congo, so the exchange between both countries is highly possible. Clade C has also been described in head lice from Ethiopia, Nepal, Pakistan, and recently in France [8, 23, 25, 37].

Four of the 86 individuals were concomitantly infested with lice belonging to clade A and C as described elsewhere [12]. One individual was infested with lice belonging to clades A and C, and only one louse of an individual belonged to clade E. This clade is more prevalent in West Africa and haplotype E46 has already been reported among head lice collected from Mali [17]. This individual could have been in close contact with a person returning from Mali and infested with head lice. Clade E had been recently described in head lice of women from DR Congo [15], but it is frequently identified in West African countries (Mali and Senegal) [17].

Among all screened bacteria, we found *A. baumannii* in 13 head lice infesting seven individuals. *Acinetobacter baumannii* has recently emerged as an important agent of nosocomial infection [38]. Its ability to acquire resistance to the main antibiotics, makes it a potentially fatal pathogen and was classified by the WHO in 2017 in the list of priority pathogens for research of new antibiotics [38, 39]. Carbapenem-resistant *A. baumannii* might be responsible for almost 60% of mortality in hospital-acquired pneumoniae and bloodstream infections [40]. Two other species of the *Acinetobacter calcoaceticus*-*A. baumannii* complex were found, *A. nosocomialis* in two head lice and *A. pittii* in one louse. Studies have shown that these species have a low prevalence rate and are less resistant and virulent than *A. baumannii* [41]. Finally, several species of the genus *Acinetobacter* have been reported in head lice collected worldwide, particularly in Africa [15, 22, 28].

We found that three of 691 lice infesting one individual were positive for a species of *Borrelia* close to *B. theileri* based on analysis of the newly generated 148-bp sequence of the *16S* gene. Our attempts to amplify the almost entire *flaB* gene of these samples failed. The low number of DNA copies of *Borrelia* spp. could explain this failure. Indeed, a study performed in Malaysia on the detection of *Borrelia* in the tick *Haemaphysalis hystricis* showed that only one of the 12 ticks detected positive by high-throughput sequencing was found to be positive by conventional PCR targeting the *16S* rRNA sequence and *flaB* gene of *Borrelia* spp. [42]. The 11 other samples positive by high-throughput sequencing showed a low relative abundance, and thus a low number of DNA copies, compared to the sample which was positive by conventional PCR. Furthermore, a previous study performed in the part of Gabon studied here found *Borrelia* spp. in blood samples of two afebrile children by qPCR targeting *16S* rRNA sequences. However, the authors failed to amplify and sequence the *flaB* gene of *Borrelia* spp., because of the low amount of DNA copies, as shown by a cycle threshold of the qPCR [43].

The presence of *Borrelia* spp. in head lice is surprising, as only *B. recurrentis* is known to be associated with louse transmission. BLAST analysis revealed that this spirochete is closest to *B. theileri*, which is known as the causative agent of cattle borreliosis [44] and has never been reported in humans. However, this species has recently been detected in head lice from Congo-Brazzaville [12]. Amanzougaghene et al. [12] argued that, since head lice are specific to humans, their infection with this bacterium may only occur during a blood meal on a human host with *B. theileri* bacteremia. This hypothesis has also been advocated in a previous study reporting the presence of *B. theileri* in head lice of pygmies, as a result of ‘accidental spill-over’ of infection in animal hosts, like those described for *B. duttoni* found in chickens and swine [12, 45].

## Conclusions

To the best of our knowledge, our study is the first to investigate simultaneously the genetic diversity of head lice and associated pathogens in Gabon. Overall, we revealed the predominance of clade A in the head lice studied. This study presents certain limits concerning the detection of pathogens, such as limited quantities of DNA for certain samples. In addition, the method of detection of *Borrelia* spp. should be optimized for small organisms such as lice, given the low sensitivity of standard PCR coupled with sequencing of the *flagellin* gene (for samples with low quantity of genomic DNA). Further investigations of the genetic diversity and bacteria associated with head lice from Gabon are needed, because data on this topic are scarce.

## Supplementary information

**Additional file 1: Table S1.** Sequences of primers and probes used in the current study.

**Additional file 2: Table S2.** Number of lice carried by patients with lice positive for *Acinetobacter baumannii* and a breakdown by patient.

## Data Availability

All data generated as well as material used during this study are included in this published article. The newly generated sequences were deposited in the GenBank database under the accession numbers: MN515370-MN515373 and MN515375-MN515380.

## Abbreviations

*cox*1: Cytochrome *c* oxidase subunit 1
*cytb*: Cytochrome *b*
DR Congo: Democratic Republic of the Congo
UDG: Uracil DNA glycosylase
BLAST: Basic Local Alignment Search Tool

## References

[1] Ascunce MS, Toups MA, Kassu G, Fane J, Scholl K, Reed DL (2013). Nuclear genetic diversity in human lice (*Pediculus humanus*) reveals continental differences and high inbreeding among worldwide populations. PLoS ONE..

[2] Ewing HE (1924). Lice from human mummies. Science..

[3] Amanzougaghene N, Mumcuoglu KY, Fenollar F, Alfi S, Yesilyurt G, Raoult D (2016). High ancient genetic diversity of human lice, *Pediculus humanus*, from Israel reveals new insights into the origin of Clade B lice. PLoS ONE..

[4] Demastes JW, Spradling TA, Hafner MS, Spies GR, Hafner DJ, Light JE (2012). Cophylogeny on a fine scale: *Geomydoecus* chewing lice and their pocket gopher hosts, *Pappogeomys bulleri*. J Parasitol..

[5] Reed DL, Light JE, Allen JM, Kirchman JJ (2007). Pair of lice lost or parasites regained: the evolutionary history of anthropoid primate lice. BMC Biol..

[6] Light JE, Toups MA, Reed DL (2008). What’s in a name: the taxonomic status of human head and body lice. Mol Phylogenet Evol..

[7] Amanzougaghene N, Fenollar F, Davoust B, Djossou F, Ashfaq M, Bitam I (2019). Mitochondrial diversity and phylogeographic analysis of *Pediculus humanus* reveals a new Amazonian clade “F”. Infect Genet Evol..

[8] Ashfaq M, Prosser S, Nasir S, Masood M, Ratnasingham S, Hebert PDN (2015). High diversity and rapid diversification in the head louse, *Pediculus humanus* (Pediculidae: Phthiraptera). Sci Rep..

[9] Ascunce MS, Fane J, Kassu G, Toloza AC, Picollo MI, González-Oliver A (2013). Mitochondrial diversity in human head louse populations across the Americas. Am J Phys Anthropol..

[10] Light JE, Allen JM, Long LM, Carter TE, Barrow L, Suren G (2008). Geographic distributions and origins of human head lice (*Pediculus humanus capitis*) based on mitochondrial data. J Parasitol..

[11] Al-Shahrani SA, Alajmi RA, Ayaad TH, Al-Shahrani MA, Shaurub ESH (2017). Genetic diversity of the human head lice, *Pediculus humanus capitis*, among primary school girls in Saudi Arabia, with reference to their prevalence. Parasitol Res..

[12] Amanzougaghene N, Akiana J, Mongo Ndombe G, Davoust B, Nsana NS, Parra HJ (2016). Head lice of pygmies reveal the presence of relapsing fever borreliae in the Republic of Congo. PLoS Negl Trop Dis..

[13] Drali R, Davoust B, Shako JC, Raoult D, Diatta G (2015). A new clade of African body and head lice infected by *Bartonella quintana* and *Yersinia pestis* - Democratic Republic of the Congo. Am J Trop Med Hyg..

[14] Louni M, Amanzougaghene N, Mana N, Fenollar F, Raoult D, Bitam I (2018). Detection of bacterial pathogens in clade E head lice collected from Niger’s refugees in Algeria. Parasit Vectors..

[15] Boumbanda Koyo CS, Amanzougaghene N, Davoust B, Tshilolo L, Lekana-Douki JB, Raoult D (2019). Genetic diversity of human head lice and molecular detection of associated bacterial pathogens in Democratic Republic of Congo. Parasit Vectors..

[16] Candy K, Amanzougaghene N, Izri A, Brun S, Durand R, Louni M (2018). Molecular survey of head and body lice, *Pediculus humanus*, in France. Vector Borne Zoonotic Dis..

[17] Amanzougaghene N, Fenollar F, Sangaré AK, Sissoko MS, Doumbo OK, Raoult D (2017). Detection of bacterial pathogens including potential new species in human head lice from Mali. PLoS ONE..

[18] Foucault C, Brouqui P, Raoult D (2006). *Bartonella quintana* characteristics and clinical management. Emerg Infect Dis..

[19] Bryceson AD, Parry EH, Perine PL, Warrell DA, Vukotich D, Leithead CS (1970). Louse-borne relapsing fever. Q J Med..

[20] Zinsser H, Grob GN. Rats, lice and history. Transaction Publishers; 2011.

[21] Kim JH, Previte DJ, Yoon KS, Murenzi E, Koehler JE, Pittendrigh BR (2017). Comparison of the proliferation and excretion of *Bartonella quintana* between body and head lice following oral challenge: immune response of human lice to *B. quintana*. Insect Mol Biol..

[22] Mana N, Louni M, Parola P, Bitam I (2017). Human head lice and pubic lice reveal the presence of several *Acinetobacter* species in Algiers, Algeria. Comp Immunol Microbiol Infect Dis..

[23] Bouvresse S, Socolovschi C, Berdjane Z, Durand R, Izri A, Raoult D (2011). No evidence of *Bartonella quintana* but detection of *Acinetobacter baumannii* in head lice from elementary schoolchildren in Paris. Comp Immunol Microbiol Infect Dis..

[24] Socolovschi C, Olive M-M, Doumbo OK, Drali R, Rogier C, Raoult D (2014). Detection of *Bartonella quintana* in African body and head lice. Am J Trop Med Hyg..

[25] Sasaki T, Poudel SKS, Isawa H, Hayashi T, Seki N, Tomita T (2006). First molecular evidence of *Bartonella quintana* in *Pediculus humanus capitis* (Phthiraptera: Pediculidae), collected from Nepalese Children. J Med Entomol..

[26] Bonilla DL, Kabeya H, Henn J, Kramer VL, Kosoy MY (2009). *Bartonella quintana* in body lice and head lice from homeless persons, San Francisco, California, USA. Emerg Infect Dis..

[27] Angelakis E, Rolain J-M, Raoult D, Brouqui P (2011). *Bartonella quintana* in head louse nits. FEMS Immunol Med Microbiol..

[28] Louni M, Mana N, Bitam I, Dahmani M, Parola P, Fenollar F (2018). Body lice of homeless people reveal the presence of several emerging bacterial pathogens in northern Algeria. PLoS Negl Trop Dis..

[29] Amanzougaghene N, Mediannikov O, Ly TDA, Gautret P, Davoust B, Fenollar F (2020). Molecular investigation and genetic diversity of *Pediculus* and *Pthirus* lice in France. Parasit Vectors..

[30] Falagas ME, Matthaiou DK, Rafailidis PI, Panos G, Pappas G (2008). Worldwide prevalence of head lice. Emerg Infect Dis..

[31] Ly TDA, Kerbaj J, Hoang VT, Louni M, Dao TL, Badiaga S (2019). The presence of *Acinetobacter baumannii* DNA on the skin of homeless people and its relationship with body lice infestation. Preliminary results. Front Cell Infect Microbiol..

[32] La Scola B, Gundi VA, Khamis A, Raoult D (2006). Sequencing of the *rpoB* gene and flanking spacers for molecular identification of *Acinetobacter* species. J Clin Microbiol..

[33] Johnson BJB, Happ CM, Mayer LW, Piesman J (1992). Detection of *Borrelia burgdorferi* in ticks by species-specific amplification of the flagellin gene. Am J Trop Med Hyg..

[34] Tamura K, Stecher G, Peterson D, Filipski A, Kumar S (2013). MEGA6: Molecular Evolutionary Genetics Analysis version 6.0. Mol Biol Evol..

[35] Librado P, Rozas J (2009). DnaSP v5: a software for comprehensive analysis of DNA polymorphism data. Bioinforma Oxf Engl..

[36] Reed DL, Smith VS, Hammond SL, Rogers AR, Clayton DH (2004). Genetic analysis of lice supports direct contact between modern and archaic humans. PLoS Biol..

[37] Angelakis E, Diatta G, Abdissa A, Trape JF, Mediannikov O, Richet H (2011). Altitude-dependent *Bartonella quintana* genotype C in head lice, Ethiopia. Emerg Infect Dis..

[38] Towner KJ (2009). *Acinetobacter*: an old friend, but a new enemy. J Hosp Infect..

[39] Tacconelli E, Magrini N, Kahlmeter G, Singh N (2017). Global priority list of antibiotic-resistant bacteria to guide research, discovery, and development of new antibiotics. WHO.

[40] Wong D, Nielsen TB, Bonomo RA, Pantapalangkoor P, Luna B, Spellberg B (2017). Clinical and pathophysiological overview of *Acinetobacter* infections: a century of challenges. Clin Microbiol Rev..

[41] Chusri S, Chongsuvivatwong V, Rivera JI, Silpapojakul K, Singkhamanan K, McNeil E (2014). Clinical outcomes of hospital-acquired infection with *Acinetobacter nosocomialis* and *Acinetobacter pittii*. Antimicrob Agents Chemother..

[42] Khoo JJ, Lim FS, Tan KK, Chen FS, Phoon WH, Khor CS (2017). Detection in Malaysia of a *Borrelia* sp. From *Haemaphysalis hystricis* (Ixodida: Ixodidae). J Med Entomol..

[43] Mourembou G, Fenollar F, Socolovschi C, Lemamy GJ, Nzoughe H, Kouna LC (2015). Molecular detection of fastidious and common bacteria as well as *Plasmodium* spp. in febrile and afebrile children in Franceville, Gabon. Am J Trop Med Hyg..

[44] McCoy BN, Maïga O, Schwan TG (2014). Detection of *Borrelia theileri* in *Rhipicephalus geigyi* from Mali. Ticks Tick Borne Dis..

[45] Cutler SJ, Abdissa A, Trape J-F (2009). New concepts for the old challenge of African relapsing fever borreliosis. Clin Microbiol Infect..

