# Supplementary material for: Molecular identification of head lice collected in Franceville (Gabon) and their associated bacteria
Source: Parasit Vectors. 2020 Aug 11;13:410. doi: 10.1186/s13071-020-04293-x (PMC7422577; doi:10.1186/s13071-020-04293-x)
Supplement: Supplementary file 1 — Additional file 1: Table S1. Sequences of primers and probes used in the current study. [file 13071_2020_4293_MOESM1_ESM.docx]

**Additional file 1: Table S1.** Sequences of primers and probes used in the current study.

| **Targets** | **Names** | **Primers (5’-3’) and probes** | **Sources** |
| --- | --- | --- | --- |
| ***P. humanus***  *Cytochrome b* | Duplex A-D | F-GATGTAAATAGAGGGTGGTT | Amanzougaghene N et al, 2016. |
|  |  | R-GAAATTCCTGAAAATCAAAC |  |
|  |  | FAM-CATTCTTGTCTACGTTCATATTTGG-TAMRA |  |
|  |  | VIC-TATTCTTGTCTACGTTCATGTTTGA-TAMRA |  |
|  | Duplex B-C/E | F-TTAGAGCGMTTRTTTACCC |  |
|  |  | R-AYAAACACACAAAAMCTCCT |  |
|  |  | FAM-GAGCTGGATAGTGATAAGGTTTAT-MGB |  |
|  |  | VIC-CTTGCCGTTTATTTTGTTGGGGTTT-TAMRA |  |
|  | *Cytb* | F-GAGCGACTGTAATTACTAATC | Li W et al, 2010. |
|  |  | R-CAACAAAATTATCCGGGTCC |  |
| ***Acinetobacter* spp*.***  RNA polymerase β subunit gene | *rpoB* | F-TACTCATATACCGAAAAGAAACGG | Bouvresse S et al, 2011. |
|  |  | R-GGYTTACCAAGRCTATACTCAAC |  |
|  |  | FAM-CGCGAAGATATCGGTCTSCAAGC-TAMRA |  |
| ***A. baumannii*** | Abau_ompA_motB F | F-TCAACATCACAATCTTTAGTAGCTGA | Ly TDA et al, 2019. |
|  | Abau_ompA_motB R1 | R-CGCTCTTGCCAGCATAAAGA |  |
|  | Abau_ompA_motB | FAM-AAGTCGCCAAGAAACCTTGA-TAMRA |  |
| ***R. prowazekii***  *rOmpB* gene | *ompB* | F-AATGCTCTTGCAGCTGGTTCT |  |
|  |  | R-TCGAGTGCTAATATTTTTGAAGCA |  |
|  |  | FAM-CGGTGGTGTTAATGCTGCGTTACAACA-TAMRA |  |
| ***Y. pestis***  Plasminogen activator gene | *PLA* | F-ATGGAGCTTATACCGGAAAC | Nguyen-Hieu T et al, 2010 |
|  |  | R-GCGATACTGGCCTGCAAG |  |
|  |  | FAM-TCCCGAAAGGAGTGCGGGTAATAGG-TAMRA |  |
| ***Borrelia* spp.**  16S ribosomal RNA | *Bor16S* | F-AGCCTTTAAAGCTTCGCTTGTAG | Parola P et al, 2011. |
|  |  | R-GCCTCCCGTAGGAGTCTGG |  |
|  |  | FAM-CCGGCCTGAGAGGGTGAACGG-TAMRA |  |
| ***B. quintana***  Hypothetical intracellular effector | *yopP* | F-TAAACCTCGGGGGAAGCAGA | Angelakis E et al, 2011. |
|  |  | R-TTTCGTCCTCAACCCCATCA |  |
|  |  | FAM-CGTTGCCGACAAGACGTCCTTG-TAMRA |  |
| ***Anaplasma* spp.**  23S ribosomal RNA | TtAna | F-TGACAGCGTACCTTTTGCAT | Dahmani M et al, 2017. |
|  |  | R-TGGAGGACCGAACCTGTTAC |  |
|  |  | FAM-GGATTAGACCCGAAACCAAG-TAMRA |  |
| ***C. burnetii***  IS1111 spacer | IS1111 | F-CAAGAAACGTATCGCTGTGGC | Mediannikov O et al, 2010. |
|  |  | R-CACAGAGCCACCGTATGAATC |  |
|  |  | FAM-CCGAGTTCGAAACAATGAGGGCTG-TAMRA |  |
