# Supplementary material for: Molecular identification of head lice collected in Franceville (Gabon) and their associated bacteria
Source: Parasit Vectors. 2020 Aug 11;13:410. doi: 10.1186/s13071-020-04293-x (PMC7422577; doi:10.1186/s13071-020-04293-x)
Supplement: Supplementary file 2 — Additional file 2: Table S2. Number of lice carried by patients with lice positive for Acinetobacter baumannii and a breakdown by patient. [file 13071_2020_4293_MOESM2_ESM.docx]

|  | **Patient ID** | **Total lice** | **Lice positive for A. baumannii** | **Percentage (%)** |
| --- | --- | --- | --- | --- |
| **1** | Patient 2 | 5 | 1 | 20.0 |
| **2** | Patient 13 | 9 | 3 | 33.3 |
| **3** | Patient 14 | 19 | 2 | 10.5 |
| **4** | Patient 28 | 2 | 1 | 50.0 |
| **5** | Patient 29 | 4 | 1 | 25.0 |
| **6** | Patient 23 | 30 | 2 | 6.7 |
| **7** | Patient 83 | 28 | 1 | 3.6 |
| **8** | Patient 86 | 30 | 2 | 6.7 |

**Additional file 2: Table S2.** Number of lice carried by patients with lice positive for *Acinetobacter baumannii* and number of lice positive for *Acinetobacter baumannii* by patient.
